# Inoculating the Public against Misinformation about Climate Change

**DOI:** 10.1002/gch2.201600008

**Published:** 2017-01-23

**Authors:** Sander van der Linden, Anthony Leiserowitz, Seth Rosenthal, Edward Maibach

**Affiliations:** ^1^ Department of Psychology University of Cambridge Cambridge UK CB2 3EB UK; ^2^ Yale Program on Climate Change Communication Yale University New Haven CT 06511 USA; ^3^ Center for Climate Change Communication George Mason University Fairfax VA 22030 USA

**Keywords:** climate change, inoculation, motivated cognition, scientific consensus

## Abstract

Effectively addressing climate change requires significant changes in individual and collective human behavior and decision‐making. Yet, in light of the increasing politicization of (climate) science, and the attempts of vested‐interest groups to undermine the scientific consensus on climate change through organized “disinformation campaigns,” identifying ways to effectively engage with the public about the issue across the political spectrum has proven difficult. A growing body of research suggests that one promising way to counteract the politicization of science is to convey the high level of normative agreement (“consensus”) among experts about the reality of human‐caused climate change. Yet, much prior research examining public opinion dynamics in the context of climate change has done so under conditions with limited external validity. Moreover, no research to date has examined how to protect the public from the spread of influential misinformation about climate change. The current research bridges this divide by exploring how people evaluate and process consensus cues in a polarized information environment. Furthermore, evidence is provided that it is possible to pre‐emptively protect (“inoculate”) public attitudes about climate change against real‐world misinformation.

## 1 Introduction

Although numerous independent assessments have found that the scientific community has reached a near‐unanimous consensus on the reality of human‐caused climate change,^1^, ^2^, ^3^, ^4^ the general public has become increasingly polarized on the issue, particularly in the United States.^5^, ^6^ This is problematic because addressing global climate change will require large‐scale changes in human behavior and decision‐making.^7^

Polarization can be amplified when the inherent uncertainty of science itself is used to cast doubt on the existence of a scientific consensus.^8^ For example, ideologically motivated, vested‐interest groups known as “Merchants of Doubt” have orchestrated influential “disinformation campaigns” in which they publicly dispute the scientific consensus on various issues, including human‐caused climate change.^9^, ^10^ These campaigns have not only successfully undermined public understanding of the degree of scientific agreement on climate change, they have also increased existing political polarization^11^ and limited deeper societal engagement with the issue.^10^, ^12^, ^13^

### 1.1 Perceived Scientific Consensus as a Gateway Cognition

One promising way to counteract the politicization of science is to highlight the strong scientific consensus about an issue when a scientific consensus exists.^8^, ^14^, ^15^ For example, a large body of research has found that “perceived scientific agreement” is a key determinant of the public's opinion on climate change.^16^, ^17^, ^18^, ^19^, ^20^, ^21^, ^22^, ^23^ In a complex and uncertain world, people often look to experts for guidance.^24^ Accordingly, research has found that in the absence of motivation to cognitively elaborate on a message, people tend to heuristically rely on consensus cues to form judgments about sociopolitical issues,^25^, ^26^, ^27^ particularly because doing so is often socially adaptive, as “consensus implies correctness.”^28^ In fact, people prefer to take cues from the combined judgment of multiple experts.^29^ As such, adopting consensus beliefs can improve judgment‐accuracy and reduce the cost of learning by condensing complex science into a simple fact (e.g., “97% of climate scientists have concluded that human‐caused global warming is happening”). At the same time, the politicization of science can undermine the influence of consensus information by triggering a motivation for (some) citizens to dismiss otherwise credible scientific evidence.^16^, ^30^ Furthermore, research finds that people's perception of expert consensus (even when correct) is easily undermined by anecdotal evidence and “false media balance,” both of which can distort the actual weight of evidence.^31^ Thus, in the face of political polarization, effectively communicating with the public about the scientific consensus requires knowledge about; (a) the way in which people attend to, process, and organize new information and (b) the structural nature of the information environment in which people form judgments and opinions about climate change.

On one hand, research has offered ample evidence for instances in which confirmation bias and motivated reasoning can lead (some) people to selectively process information and reject evidence that runs contrary to prior beliefs or deeply held ideological worldviews.^32^, ^33^, ^34^, ^35^, ^36^ For example, the cultural cognition thesis predicts that conveying scientific agreement about contested societal issues will only increase attitude polarization.^37^

On the other hand, scholars have questioned the validity of the cultural cognition thesis,^38^, ^39^ especially because the biased assimilation of information is just one of many ways by which people can orient themselves toward science and the environment.^40^ A substantial body of research has found that communicating the degree of scientific agreement on contested societal issues, such as vaccines and climate change, can shift public perception of the scientific consensus, which in turn influences other key beliefs, such as the belief that climate change is happening, human‐caused, and a serious issue that requires public action.^7^, ^41^

People's subjective perceptions about what other groups believe (i.e., “metacognitions”) often serve as informational judgment cues. Accordingly, many studies find that conveying the fact that most scientists are convinced that human‐caused climate change is happening can increase perceived consensus and acceptance of anthropogenic climate change across the ideological spectrum, either directly or indirectly.^19^, ^42^, ^43^, ^44^ In particular, the gateway belief model (GBM) developed by van der Linden et al.^7^ suggests that reducing the “gap” between people's subjective perception and the actual of level of normative agreement among influential referents (e.g., experts) can lead to small yet important changes in other key personal beliefs. Indeed, much social–psychological research has shown that debiasing people's perception of the norm often has a positive cascading effect on other personal beliefs and behaviors.^45^, ^46^ Yet, although highlighting scientific consensus can neutralize polarizing worldviews^19^, ^44^ and reduce motivated reasoning,^8^ more mixed evidence has also been noted.^47^

### 1.2 Countering the Spread and Influence of Misinformation: Inoculation Theory

More generally, people often process conflicting informational cues at the same time.^48^ Thus, although highlighting scientific agreement has been found effective under stylized conditions, its efficacy in the presence of real‐world misinformation remains unclear.^49^, ^50^ Yet, evaluating this is important because the pairing of conflicting informational cues is an explicit opportunity to examine motivated cognition. To our knowledge, no research to date has examined if and how public beliefs about the scientific consensus on climate change are affected by, or can be protected against, “sticky” misinformation. In fact, researchers have recently conceptualized the process by which misinformation spreads through a population as a metaphorical “contagion.”^51^ A closely related term is a “meme,” which is often described as an idea, behavior, or style that spreads from person to person within a culture.^52^, ^53^ In the context of global warming, a false meme can be thought of as an inaccurate mental belief (e.g., there is no consensus among climate scientist) that is transmitted (replicated) from one mind to another.^54^ Because of their socially infectious nature, (false) memes are sometimes referred to as “thought contagions.”^55^

The rate of cultural transmission, or infection, may be slowed through a process known as attitudinal inoculation. In medicine, resistance to a virus can be conferred by exposing someone to a weakened version of the virus (a vaccine)—strong enough to trigger a response (i.e., the production of antibodies), but not so strong as to overwhelm the body's immune system. The social–psychological theory of attitudinal inoculation^56^ follows a similar logic: A threat is introduced by forewarning people that they may be exposed to information that challenges their existing beliefs or behaviors. Then, one or more (weakened) examples of that information are presented and directly refuted in a process called “refutational pre‐emption” or “prebunking.”^14^ In short, attitudinal resistance is conferred by pre‐emptively highlighting false claims and refuting potential counterarguments.

Although a large body of research on inoculation theory has demonstrated its efficacy^57^ in a variety of applied contexts, most notably in the areas of health^58^ and political campaigning,^59^ inoculation theory has not been tested in the context of climate change. Moreover, prior inoculation theory research has primarily examined how positive attitudes toward simple “cultural truisms” can be maintained.^60^ Yet, there are many issues, including climate change, where people have strongly differing pre‐existing (political) attitudes. Accordingly, this study addresses the following two key research questions: (1) does the presence of misinformation “negate” the positive effect of communicating the scientific consensus on climate change? And if so, (2) is it possible to “inoculate” public attitudes about the degree of scientific consensus against (influential) misinformation? Drawing on prior research, we hypothesize that the process of inoculation will indeed protect pre‐existing (positive) attitudes as well as help counteract motivated reasoning.

## 2 Method

Two studies were conducted to answer these research questions. In the first study, we used a nationally representative probability sample of the US population (*N* = 1000) to test several misinformation statements about the scientific consensus on human‐caused climate change. The purpose of Study 1 was to identify the most influential and representative “countermessages” used by climate change opponents. In Study 2, we conducted a randomized online survey experiment using a large and diverse sample (*N* = 2167) from Amazon Mechanical Turk (Mturk) to test whether it is possible to “inoculate” people against such misinformation (see Part B in the Supporting Information for more information about Mturk). We employed a mixed design that compared a participant's pre–post (within‐subject) estimate of the scientific consensus across (between) six different experimental conditions. An overview of the different experimental conditions is provided in **Table** **1**.

**Table 1:** Overview of experimental conditions

Experimental treatment conditions
1. Control group
2. Consensus (“pie chart”) treatment (CT)
3. Countermessage (CM)
4. Consensus‐treatment followed by countermessage (CT | CM)
5. Consensus‐treatment + general inoculation followed by countermessage (In1 | CM)
6. Consensus‐treatment + detailed inoculation followed by countermessage (In2 | CM)

In short, we hypothesized that communicating the scientific consensus (by itself) would have a positive influence on perceived scientific agreement (condition 2), whereas the countermessage (by itself) would have a negative impact (condition 3). We also hypothesized that the presence of counterinformation would diminish the general efficacy of the consensus message (condition 4). Finally, as a direct test of inoculation, we hypothesized that both a general and more specific inoculation message would protect the consensus‐treatment against the misinformation statement (conditions 5 and 6). Participants in the control group (condition 1) solved a neutral word puzzle.

Study 1 investigated which countermessage was most influential with the American public. Six common statements were tested (see Part A in the Supporting Information for a full description of the study). Respondents ranked each statement on two dimensions: familiarity and persuasiveness. Out of all statements, respondents were most familiar with and convinced by the argument that “there is no consensus on human‐caused climate change.” This argument was based on a real disinformation campaign (“The Oregon Global Warming Petition Project,” 2007)^61^ which hosts a website claiming that; “over 31 000 American scientists have signed a petition stating that there is no scientific evidence that the human release of carbon dioxide will, in the foreseeable future, cause catastrophic heating of the Earth's atmosphere.” An exact copy of the petition was used as the main countermessage in Study 2, but all identifying source‐information was redacted to prevent confounding effects between the source and the message.

Prior research has found that the scientific consensus is effectively communicated in the form of a pie chart stating: “97% of climate scientists have concluded that human‐caused climate change is happening.”^44^ To ensure a representative study design in which the messages shown to participants reflect real‐world content, we mimicked the design of the pie chart used by the “Consensus Project”^62^—because this graphic has frequently been featured in the media. The inoculation messages consisted of two components: (a) warning of an impending threat/attack on one's prior beliefs and attitudes (affective component) and (b) a pre‐emptive refutation (cognitive component). In the shorter, general version, respondents were first warned: “some politically motivated groups use misleading tactics to try to convince the public that there is a lot of disagreement among scientists.” This claim was then debunked by reiterating that scientific research has found that among climate scientists, there is virtually no disagreement that humans are causing climate change. In the longer, more specific inoculation condition, additional arguments were added to debunk the Oregon Petition specifically (e.g., by highlighting that some of the signatories are fraudulent, including Charles Darwin and members of the Spice Girls, that fewer than 1% of the signatories have a background in atmospheric/climate science, etc.).

The design of the experiment follows a linear‐additive format (Table 1)—i.e., in both the consensus‐ and countermessage‐only conditions, respondents only read the relevant message in isolation. In the general inoculation condition, respondents first read the consensus statement, followed by a general inoculation, before being exposed to the countermessage. In the detailed inoculation condition, respondents were first shown the consensus message, followed by the general inoculation and then the more detailed inoculation message, before being exposed to the countermessage. This design allowed us to assess the marginal benefit of the (two) inoculation strategies. A full description of all treatments used in Study 2 is provided in part B of the Supporting Information.

The main dependent variable is a respondent's (pre and post) estimate of the current level of scientific agreement on human‐caused climate change (0%–100%). In addition, subjects were also asked how certain they are about their estimate, how likely they think it is that climate change is happening, whether they believe it is human‐caused, how much they worry about the issue, and whether people should be doing more or less about climate change. To disguise the true purpose of the experiment, participants were told that they would randomly be asked about 1 out of 20 possible media topics (the topic was always the same). All subjects were presented with the same question set before (pre) and after (post) the treatments were administered. A manipulation check was also included to verify the efficacy of the treatment effects. An overview of the sample characteristics (and census data for comparison purposes) is provided in **Table** **2**. A description of the MTurk procedure and platform is provided in Part B of the Supporting Information.

**Table 2:** Sample characteristics

Sample	(*N* = 2167)	Census
Demographic characteristics		
Gender (% female)	56	51
Age 18–65+ (modal bracket)	25–44	38
Education (% college degree or higher)	50	32
Region (% Northeast)	17.3	17.7
Party affiliation (% Democrat)	37	32

Note: US population 2013 census estimates. Age (median). Political party affiliation estimate by Pew (2013).

## 3 Results

**Figure 1:**
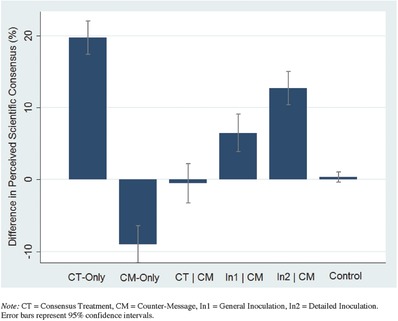
Overview of mean (pre–post) differences in perceived scientific consensus by treatment group. Note: CT = consensus treatment, CM = countermessage, In1 = general inoculation, In2 = detailed inoculation. Error bars represent 95% confidence intervals.

All of the hypotheses were fully supported by the data. Descriptive within‐subject differences in perceived scientific agreement are reported in **Table** **3** and **Figure** **1**. As expected, no meaningful pre–post change in perceived consensus was observed in the control group (*M*_diff_ = 0.35). The consensus‐treatment (CT) alone elicited a large increase in perceived scientific agreement (*M*_diff_ = 19.72). In contrast, the (misinformation) countermessage (CM) had a substantial negative influence (*M*_diff_ = −8.99) when presented on its own. When participants viewed the messages sequentially (CT | CM), the informational value of the consensus‐treatment was negated completely (*M*_diff_ = 0.51). As hypothesized, the general (In1 | CM) and detailed (In2 | CM) inoculation interventions were each successful in preserving much of the positive effect of the consensus message in the presence of counterinformation (*M*_diff_ = 6.47 and 12.71—or one‐third and two‐thirds of the initial consensus‐treatment effect, respectively).

**Table 3:** Descriptive overview of mean (pre–post) differences in perceived scientific consensus by treatment group

Treatment conditions	Perceived scientific consensus [%] (pretest mean)	Perceived scientific consensus [%] (post‐test mean)	Difference (post‐pretest) (standard error)	Cohen's D (vs control)
Control group (*n* = 360)	72.18	72.53	0.35 (0.36)	–
Consensus‐treatment (CT) (*n* = 338)	70.58	90.30	19.72 (1.17)	1.23
Countermessage (CM) (*n* = 392)	72.04	63.05	−8.99 (1.31)	0.48
Consensus‐treatment (CT) | CM (*n* = 352)	73.48	72.99	−0.51 (1.39)	0.04
CT + general inoculation | CM (*n* = 363)	73.29	79.76	6.47 (1.32)	0.33
CT + detailed inoculation | CM (*n* = 362)	71.23	83.94	12.71 (1.17)	0.75

To test whether these differences are statistically significant, an analysis of covariance (ANCOVA) was conducted with the post‐test as the dependent variable and the pretest as the covariate. The ANCOVA revealed a significant main effect for the treatment groups, *F*(5, 2160) = 82.10, mean squared error (MSE) = 443.92, *p* < 0.001, $\eta_{p}^{2}=0.16$. Post hoc comparisons on the adjusted marginal means using the Tukey honest significant difference (HSD) test indicated significant between‐group differences for all the previously stated comparisons (*p* < 0.001). There was one exception, as expected, the difference between the control group ($\bar{x}=0.35$, standard error (SE) = 0.36) and the “neutralizing” (CT | CM) condition ($\bar{x}=0.51$, SE = 1.39) was not significant.

A number of manipulation checks were performed to assess the consistency of the results. At the end of the survey, participants were asked, using a seven‐point scale, to indicate how convincing they found the experimental treatments. Respondents who only viewed the consensus‐treatment thought the message was significantly more convincing than those who viewed the consensus message in the presence of counterinformation ($\bar{x}=5.11$ vs $\bar{x}=4.80$), *t*(1237) = 2.42, *p* < 0.01. Similarly, participants who viewed the counterinformation by itself thought it was significantly more convincing than when viewed in the presence of the consensus‐treatment ($\bar{x}=3.71$ vs $\bar{x}=3.26$), *t*(1037) = 3.13, *p* < 0.01. In a similar vein, respondents who found the consensus‐treatment more convincing (median split) adjusted their estimate of the scientific consensus at a higher rate than those who were less convinced ($\bar{x}=8.18>\bar{x}=5.86$), *t*(1526) = 1.72, *p* < 0.05).

Next, within‐subject differences in perceived consensus were examined for each treatment condition by political party identification (**Table** **4**). On the whole, the pattern is strikingly similar across party lines, i.e., the consensus‐treatment on its own elicits the greatest change, the countermessage by itself has a negative effect, sequential messaging neutralizes the positive effect of the consensus‐treatment while the general and specific inoculation conditions both successfully preserve similar proportions of the treatment‐effect across political party affiliation.

**Table 4:** Descriptive overview of mean (pre–post) differences in perceived scientific consensus by political party affiliation

Treatment conditions	Democrat (*n* = 788)	Independent (*n* = 646)	Republican (*n* = 390)
Control group	0.74	−0.67	1.90
Consensus‐treatment (CT)	15.78	19.05	23.00
Countermessage (CM)	−9.11	−8.50	−9.03
Consensus‐treatment (CT) | CM	0.57	1.61	−8.03
CT + general inoculation | CM	4.48	6.97	6.92
CT + detailed inoculation | CM	11.08	12.79	10.75

**Figure 2:**
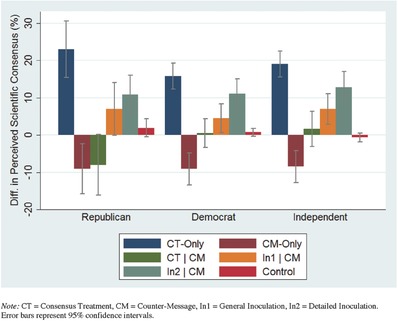
Overview of mean (pre–post) differences in perceived scientific consensus by political party affiliation. Note: CT = consensus treatment, CM = countermessage, In1 = general inoculation, In2 = detailed inoculation. Error bars represent 95% confidence intervals.

Yet, two observed differences are noteworthy. First, the effect of the consensus‐only treatment is somewhat larger (descriptively) for Republicans (*M*_diff_ = 23.00) and Independents (*M*_diff_ = 19.05) compared to Democrats (*M*_diff_ = 15.78). Second, while the presence of misinformation “neutralizes” the effect of the consensus‐treatment for both Democrats (*M*_diff_ = 0.57) and Independents (*M*_diff_ = 1.61), it has a negative effect on Republican respondents (*M*_diff_ = −8.03). In other words, on average, only Republicans reduced their consensus estimates when they viewed the consensus message followed by the counterinformation. Accordingly, an ANCOVA revealed a small but significant interaction between the treatment conditions and political party, *F*(11, 1805) = 2.12, MSE = 415.77, *p* = 0.02, $\eta_{p}^{2}=0.01$). Main results are presented in Table 4 and **Figure** **2**.

A main effect was also found for belief certainty, *F*(5, 2160) = 52.94, MSE = 2.09, *p* < 0.01, $\eta_{p}^{2}=0.11$. Post hoc comparisons using the Tukey Honest Significant Difference (HSD) test indicated that the consensus‐treatment and inoculation conditions significantly increased belief certainty of consensus estimates compared to the counterinformation and control groups (*p* < 0.01). On average, the consensus‐treatment (by itself) increased belief certainty (1–7 scale) by (*M*_diff_ = 1.63, SE = 0.11) versus (*M*_diff_ = 0.62, SE = 0.09) in the presence of counterinformation. As expected, much of the initial effect was preserved in both the general and more detailed inoculation conditions (*M*_diff_ = 0.90, SE = 0.09 and *M*_diff_ = 1.21, SE = 0.10). Finally, the treatments did not have notable main effects on other key beliefs about climate change—with the exception of normative support for public action, *F*(5, 2160) = 13.54, MSE = 0.41, *p* < 0.01, $\eta_{p}^{2}=0.03$. Compared to the control group (*p* < 0.05), the consensus‐message (by itself) had a small positive main effect ($\bar{x}=0.14$, SE = 0.05).^63^

## 4 Discussion

This study finds that public attitudes about climate change can be effectively “inoculated” against influential misinformation. In particular, our results point to three important conclusions. First, consistent with prior work, we find strong support for the efficacy of communicating the scientific consensus on human‐caused climate change.^7^, ^19^, ^38^, ^43^, ^44^, ^47^ Second, this research further extends these findings by presenting information about the consensus in a politically “contested” information environment, that is, countered by a real petition claiming that there is no scientific consensus on human‐caused climate change. As such, we help address the criticism that prior experiments “do not realistically model the real‐world dynamics of opinion formation relevant to the climate change dispute” (ref. 49], p. 16).

Results indicate that the positive influence of the “consensus message” is largely negated when presented alongside such misinformation. Thus, in evaluating the efficacy of consensus messaging, scholars should recognize the potent role of misinformation in undermining real‐world attempts to convey the scientific consensus. Third, the current study also found that much of the initial consensus‐effect was preserved (up to two‐thirds) by the inoculation messages, which, importantly, proved equally effective across the political spectrum. Accordingly, “inoculation” is a promising approach to protect public understanding of the extant scientific consensus that human‐caused climate change is happening, which, as prior research has shown, acts as an important “gateway” cognition to other keys beliefs about the issue.^7^, ^17^

Some scholars have argued that because people sometimes engage in “identity‐protective motivated reasoning,” highlighting scientific consensus will only cause or exacerbate existing attitude polarization.^37^ Yet, “true” attitude polarization in response to mixed evidence is relatively infrequent (ref. 64, 65, 66 and recent research suggests that political polarization on climate change is more likely the result of selective exposure to partisan media rather than motivated reasoning alone.^48^, ^50^, ^67^, ^68^ Moreover, this study finds no support for the hypothesis that inoculating people about the scientific consensus backfires among those who are ideologically predisposed to be skeptical about climate change (e.g., Republicans), which is both promising and consistent with other research on inoculation theory (e.g., see ref. 60). In fact, we extend inoculation research in a novel direction by testing its efficacy in the context of a highly politicized issue.

This is not to say that the motivated processing of political information does not occur.^33^ For example, simple corrections can backfire among the targeted ideological group.^69^ Other recent research has suggested that communicating the scientific consensus on climate change may backfire among strong “free‐market” endorsers.^47^ Similarly, we find that when the consensus and countermessages were presented sequentially, Republican respondents were, on average, indeed more likely to weigh the “no consensus” treatment more heavily in their subsequent judgment of the scientific consensus. Yet, it is important to note that even in this case, highlighting scientific agreement is still beneficial, as the magnitude of the observed “negative effect” among Republicans is actually less (or at the very least, no different) from what it would have been if no consensus information had been presented at all. In other words, Republican respondents who only saw the countermessage decreased their estimate of the scientific consensus more than Republican respondents who saw both messages. Thus, we find no evidence that conveying strong normative agreement among experts “backfires” with potentially skeptical audiences.^70^

More importantly, both inoculation messages proved effective in protecting the positive effect of the consensus message and shifted the opinions of Republicans, Independents, and Democrats alike in a direction consistent with the conclusions of climate science. Moreover, these results are consistent with other recent research, which has also found that warning people pre‐emptively of counterattitudinal messages can help reduce directional motivated reasoning (e.g., see ref. 8).

Practically, these findings suggest that, when possible, communicating the scientific consensus on human‐caused climate change should be accompanied by information that forewarns the public that politically or economically motivated actors may seek to undermine the findings of climate science. In addition, audiences should be provided with the “cognitive repertoire”—a basic explanation about the nature of disinformation campaigns— to pre‐emptively refute such attempts. In short, these findings add to a growing body of research reporting that communicating a social fact, such as the high level of agreement among experts about the reality of human‐caused climate change, can be an effective and depolarizing public engagement strategy.^7^, ^19^, ^43^, ^44^, ^47^

Finally, this study is of course not without limitations. First, we were unable to assess the rate of decay (if any) of the effect of the inoculation messages. However, other recent research has indicated that the positive effects of attitudinal inoculation do persist over time (e.g., ref. 71), although more longitudinal research is needed. Second, while great care was taken to ensure a representative design, laboratory research is limited in its ability to simulate the structure of an individual's information environment. Thus, we look forward to and encourage future research to test and extend these findings in real‐world (field) settings.

## 5 Conclusion

In a large experiment (*N* = 2167), we show that communicating the scientific consensus on human‐caused climate change significantly increases public perception of the expert consensus by about 20 percentage points (Bar I, CT‐Only). Importantly, the introduction of (mis)information contesting the existence of a scientific consensus neutralizes the positive effect of highlighting normative expert agreement (Bar III, CT|CM). Further, in the absence of any cues about the actual level of consensus, the presentation of misinformation significantly undermines the public's perception of the level of scientific agreement (−9 points; Bar II, CM). Finally, pre‐emptively warning people about politically motivated attempts to spread misinformation helps promote and protect (“inoculate”) public attitudes about the scientific consensus (Bars IV and V, In1 | CM and In2 | CM).

## Supporting information

As a service to our authors and readers, this journal provides supporting information supplied by the authors. Such materials are peer reviewed and may be re‐organized for online delivery, but are not copy‐edited or typeset. Technical support issues arising from supporting information (other than missing files) should be addressed to the authors.

Supplementary
