# Supplementary material for: Inoculating the Public against Misinformation about Climate Change
Source: Glob Chall. 2017 Jan 23;1(2):1600008. doi: 10.1002/gch2.201600008 (PMC6607159; doi:10.1002/gch2.201600008)
Supplement: Supplementary file 1 — Supplementary [file GCH2-1-1600008-s001.pdf]

Copyright WILEY-VCH Verlag GmbH & Co. KGaA, 69469 Weinheim, Germany, 2017.

Global Challenges

Open Access

Supporting Information

for *Global Challenges*, DOI: 10.1002/gch2. 201600008

# Inoculating the Public against Misinformation about Climate Change

*Sander van der Linden,* Anthony Leiserowitz, Seth Rosenthal, and Edward Maibach*

## SUPPLEMENTARY INFORMATION A

Data for Study I was gathered between the 13th and 15th of June, 2014 by the OmniWeb KnowledgePanel, which is a national online omnibus service of GfK Research North America. We obtained a nationally representative probability sample of the US population ($N$ = 1,000) to test the public's familiarity with and (perceived) persuasiveness of the following six (misinformation) statements about climate change:

1. **There is no consensus among scientists about climate change**. Although some people claim that most climate scientists say human-caused global warming is happening, this information is simply not true. In fact, over *31,000* scientists have signed a petition stating: "There is no convincing scientific evidence that the human release of carbon dioxide will, in the foreseeable future, cause catastrophic heating of the Earth's atmosphere" ($M$ = 6.11, $SE$ = 0.20).

2. **Most climate scientists say human-caused global warming is happening because that's how they get government grant money**. Although most climate scientists say human-caused global warming is happening, that's only because that's the only way they can get big government grants to fund themselves. To quote Texas governor Rick Perry, "There are a substantial number of scientists who have manipulated data so that they will have dollars rolling into their projects" ($M$ = 5.36, SE = 0.19).

3. **Global warming is not real, therefore the "consensus" is a hoax.** The Earth's climate changes naturally. The claim that it is warming due to human activities is a hoax advanced by foreign governments, the United Nations, and scientists on their payroll, in an attempt to scare and manipulate the public. The so-called "scientific consensus" that 97% of climate scientists say that human-caused global warming is happening – is also a hoax. According to climate scientist

and former University of Winnipeg professor Tim Ball, Ph.D., “it is the greatest deception in history and the extent of the damage has yet to be exposed and measured” (*M* = 5.59, SE = 0.18).

4. **<u>The so-called “consensus” studies showing that climate scientists agree human-caused global warming is happening are flawed.</u>** Studies claiming that 97% of climate scientists say human-caused global warming is happening are flawed because they purposefully exclude scientists who disagree, as Director of the Global Warming Policy Foundation, Benny Peiser, Ph.D., concluded from his own investigation: “Consensus? What consensus?” Dr. Peiser found that a substantial number of studies publicly reject the consensus position (*M* = 5.33, SE = 0.18).

5. **<u>The IPCC is an alarmist organization, filled with alarmist scientists who are creating a “false consensus” about the reality of human-caused climate change.</u>** The U.N.’s Intergovernmental Panel on Climate Change (IPCC) is an alarmist organization, led by a small group of politically-motivated scientists who distort the evidence and have manufactured a “scientific consensus” on human-caused global warming to make it sound more dangerous than it actually is. In fact, economics Professor Richard Tol, Ph.D., of the University of Sussex resigned from the IPCC because he felt that their latest report was “too alarmist” (*M* = 4.17, SE = 0.15).

6. **<u>Climate scientists are doing everything possible to silence the global warming skeptics.</u>** Although most climate scientists say human-caused global warming is happening, many do not. Unfortunately, throughout history scientists that have questioned official dogma have often been ignored, mocked or even forced into silence, even when they were right. For example, Galileo was forced to publicly retract his finding that the Earth revolves around the sun because it went against the teachings of the Catholic Church. Today, the same thing is happening to scientists who dare to question the reality of human-caused global warming (*M* = 6.04, SE = 0.20).

## SUPPLEMENTARY INFORMATION B

Data for Study 2 (*N* = 2,163) was gathered on the Amazon Mechanical Turk (MTurk) platform. Although it was important to verify that the "misinformation" treatments were actually representative of national opinion in the initial study, for the second study, we focused on the internal validity of the experimental results rather than on generalizing public opinion. Nonetheless, a wide variety of assessments have now shown that Mturk is a reliable platform for experimental data collection and that Mturk samples are more demographically diverse than other forms of convenience, student, or internet-based samples, especially in terms of political ideology (Berinsky et al., 2012; Buhrmester et al., 2011; Clifford et al., 2015; Goodman et al., 2013). Participants were invited to participate in a public opinion study about climate change and paid $0.50 cents for the task. The approval rate for participation was set greater than or equal to 95%. Location was restricted to the United States. We used Qualtrics for the survey design. Ethical approval for both studies was obtained from the Yale Institutional Review Board (IRB).

### REFERENCES


Berinsky, A.J., Huber, G.A., & Lenz, G.S. (2012). Evaluating online labor markets for experimental research: Amazon. com's Mechanical Turk. *Political Analysis*, *20*(3), 351-368.

Buhrmester, M., Kwang, T., & Gosling, S. D. (2011). Amazon's Mechanical Turk: A new source of inexpensive, yet high-quality, data? *Perspectives on Psychological Science*, *6*(1), 3-5.

Clifford, S., Jewell, R.M., & Waggoner, P.D. (2015). Are samples drawn from Mechanical Turk valid for research on political ideology? *Research & Politics*, *2*(4), 1-9.

Goodman, J.K., Cryder, C.E., & Cheema, A. (2013). Data collection in a flat world: The strengths and weaknesses of Mechanical Turk samples. *Journal of Behavioral Decision Making*, *26*(3), 213-224.

## MEASUREMENT

### Estimate of the scientific consensus

Subjects were asked the following question: “To the best of your knowledge, what percentage of climate scientists have concluded that human-caused climate change is happening?” Response options were given on a continuum (slider-scale), ranging from 0% of climate scientists think that human-caused climate change is happening to 100%.

### Belief certainty of estimate

Directly following estimates of the scientific consensus, subjects were asked how certain they are about their answer. Response options were given on a continuum, ranging from 1 (I am not at all certain), 4 (neutral) to 7 (I am very certain).

### Belief in Climate Change

Subjects were asked the following question; “What do you think; Do you think that climate change is happening or, not?” Response options included; 1 (I think climate change is happening), 2 (I think climate change is NOT happening) or 3 (I am unsure whether or not climate change is happening).

### Human-Causation

Subjects who indicated that they think climate change is happening, or are unsure about whether or not climate change is happening, were asked a follow-up question; “Which of the statements below best describes your opinion?” Response options included;
1 (Climate change is caused entirely by human activities), 2 (Climate change is caused mostly by human activities), 3 (Climate change is caused equally by human activities and

natural changes in the environment), 4 (Climate change is caused mostly by natural changes in the environment) or 5 (Climate change is caused entirely by natural changes in the environment) - an extra option was reserved for “I don’t know”.

### Worry about Climate Change

Subjects were asked the following question; “On a scale from 1 to 7, how worried are you about climate change?” Response options were given on a continuum, ranging from 1 (I am not at all worried), 4 (neutral) to 7 (I am very worried).

### Public Action

Subjects were asked the following question; “Do you think people should be doing more or less to reduce climate change?” Response options were given on a continuum, ranging from 1 (much less), 4 (same amount) to 7 (much more).

### Manipulation/Attention check(s)

At the end of the survey, participants were first asked whether they had seen a consensus or counter-message statement (yes, no, don’t remember). Following the attention check, subjects were asked how much they *agreed* with the respective statement 1 (completely disagree), 4 (neither agree nor disagree) to 7 (completely agree) and how *convincing* they found the statement 1 (very unconvincing), 4 (neutral) 7 (very convincing).

**SUPPLEMENTARY INFORMATION C**

Examples of the experimental treatment conditions are provided below:

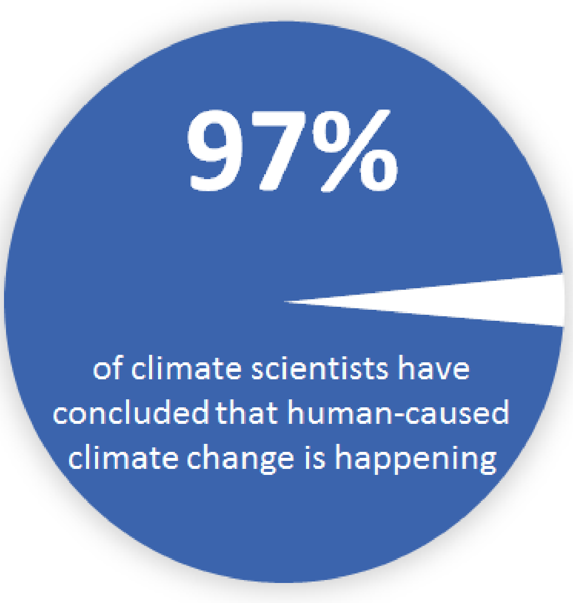


**Treatment Example 1**: Consensus Message (Only)

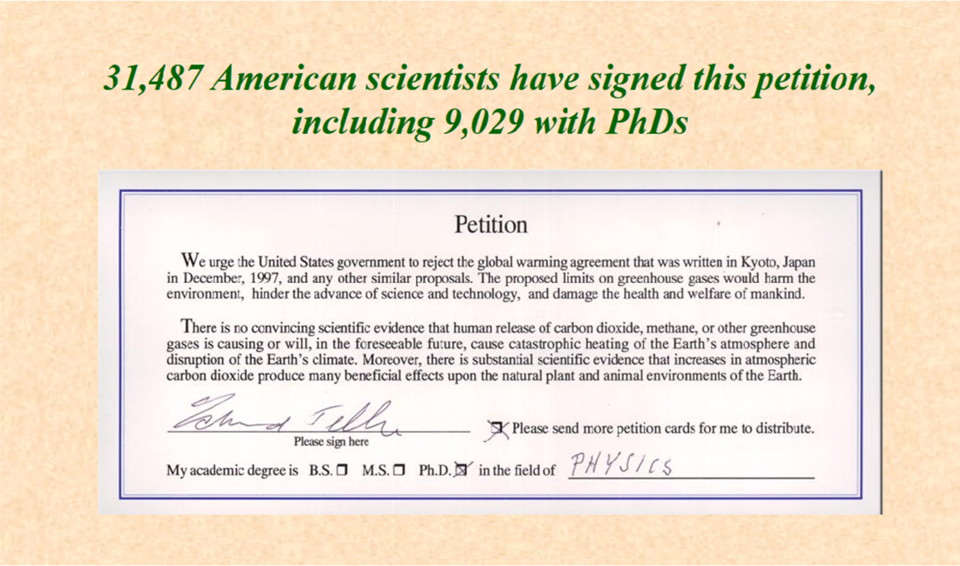

*31,487 American scientists have signed this petition, including 9,029 with PhDs*

Petition

We urge the United States government to reject the global warming agreement that was written in Kyoto, Japan in December, 1997, and any other similar proposals. The proposed limits on greenhouse gases would harm the environment, hinder the advance of science and technology, and damage the health and welfare of mankind.

There is no convincing scientific evidence that human release of carbon dioxide, methane, or other greenhouse gases is causing or will, in the foreseeable future, cause catastrophic heating of the Earth's atmosphere and disruption of the Earth's climate. Moreover, there is substantial scientific evidence that increases in atmospheric carbon dioxide produce many beneficial effects upon the natural plant and animal environments of the Earth.

[illegible] Please sign here ☒ Please send more petition cards for me to distribute.

My academic degree is B.S. ☐ M.S. ☐ Ph.D. ☒ in the field of PHYSICS

**Treatment Example 2**: Counter-Message (Only)

**Treatment Example 3:** General (In1) and Detailed (In2) Inoculation Messages

General: Nearly all climate scientists—97%—have concluded that human-caused climate change is happening. Some politically-motivated groups use misleading tactics to try to convince the public that there is a lot of disagreement among scientists. However, scientific research has found that among climate scientists "there is virtually no disagreement that humans are causing climate change".

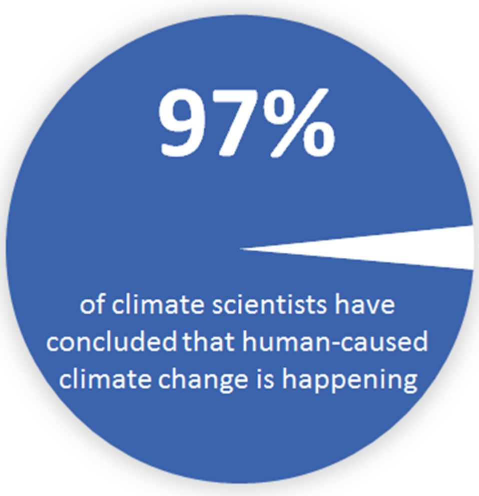


Detailed: One such politically motivated group claims to have collected signatures from over 31,000 "scientists" (including over 9,000 who hold Ph.D.'s) on a petition urging the U.S. government to reject any limits on greenhouse gas emissions because; "there is no convincing scientific evidence that human release of carbon dioxide, methane or other greenhouse gases is causing or will, in the foreseeable future, cause catastrophic heating of the Earth's atmosphere and disruption of Earth's climate." They claim that these signatures prove that there is no scientific consensus on human-caused climate change.

This may sound convincing at first. However, several independent investigations have concluded that the "Petition Project" is extremely misleading. For instance, many of the signatures on the petition are fake (for example, past signatories have included the long-deceased Charles Darwin, members of the Spice Girls, and fictional characters from Star Wars). Also, although 31,000 may seem like a large number, it actually represents less than 0.3% of all US science graduates (a tiny fraction). Further, nearly all of the legitimate signers have no expertise in climate science at all. In fact, less than 1% of those who signed the petition claim to have any background in Climate or Atmospheric Science. Simply calling yourself a "scientist" does not make someone an expert in climate science. By contrast, 97% of actual climate scientists, agree that human-caused climate change is happening.

**SUPPLEMENTARY INFORMATION D**

*Figure 3.* Overview of Mean (Pre-Post) Differences in Perceived Scientific Consensus by Prior Attitude Toward Climate Change.

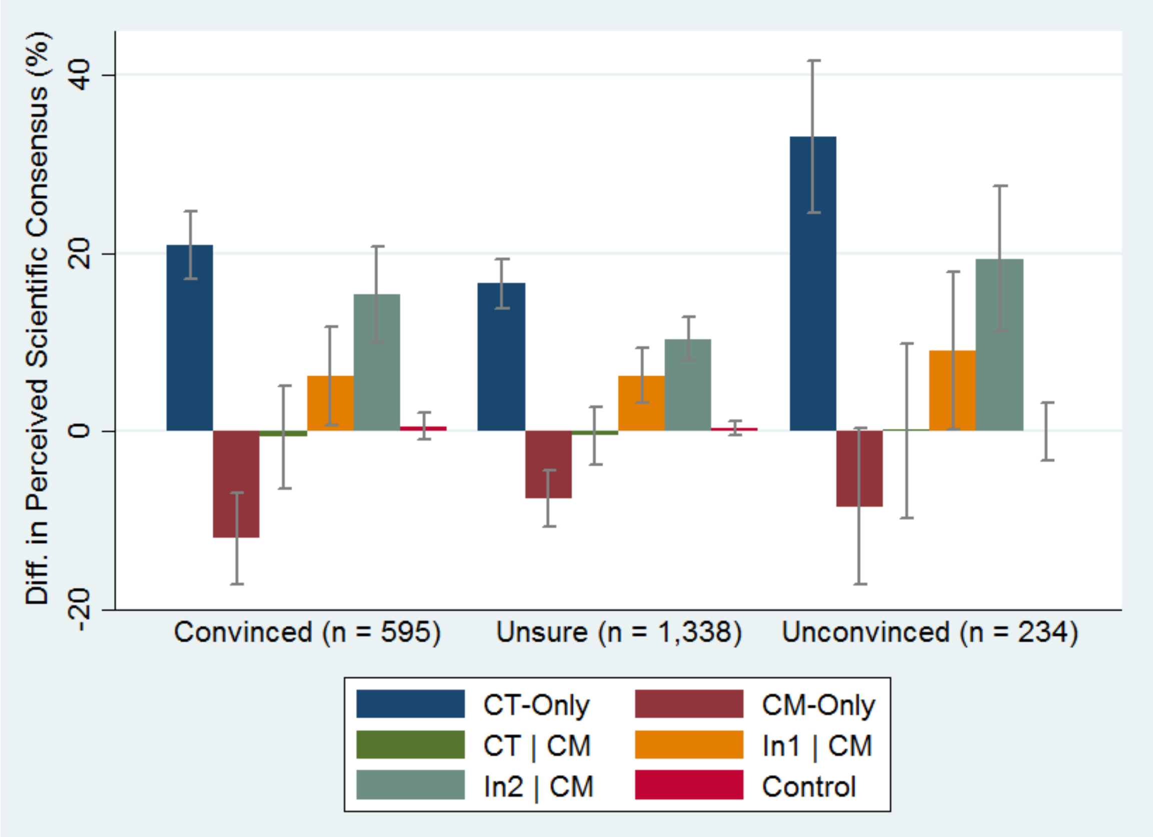


*Note:* CT = Consensus Treatment, CM = Counter-Message, In1 = General Inoculation, In2 = Detailed Inoculation. Error bars represent 95% confidence intervals. The three attitudinal groups were created based on combinations of answers to the pre-test questions, such that those respondents who answered that they believe that climate change is happening AND human-caused were classified as “convinced”, those who stated that they do not believe that climate change is happening at all were classified as “unconvinced” and the remainder of the sample was classified as “unsure” (e.g., those respondents who are unsure about whether or not climate change is happening, or those who believe that climate change is happening but are unsure as to whether it is human-caused).
